# Shedding light on pediatric diseases: multispectral optoacoustic tomography at the doorway to clinical applications

**DOI:** 10.1186/s40348-020-00095-4

**Published:** 2020-03-04

**Authors:** Adrian P. Regensburger, Alexandra L. Wagner, Jing Claussen, Maximilian J. Waldner, Ferdinand Knieling

**Affiliations:** 1 Pediatric Experimental and Translational Imaging Laboratory (PETI-Lab), Department of Pediatrics and Adolescent Medicine, Friedrich-Alexander-University (FAU) Erlangen-Nuremberg, Loschgestraße 15, 91054 Erlangen, Germany; 2 Medical Department 1, Friedrich-Alexander-University (FAU) Erlangen-Nuremberg, Erlangen, Germany

## Abstract

Optoacoustic imaging (OAI), or photoacoustic imaging (PAI), has fundamentally influenced basic science by providing high-resolution visualization of biological mechanisms. With the introduction of multispectral optoacoustic tomography (MSOT), these technologies have now moved closer to clinical applications. MSOT utilizes short-pulsed near-infrared laser light to induce thermoelastic expansion in targeted tissues. This results in acoustic pressure waves, which are used to resolve specific endo- and exogenous chromophores. Especially in the pediatric population, this non-invasive imaging approach might hold fundamental advantages compared to conventional cross-sectional imaging modalities. As this technology allows the visualization of quantitative molecular tissue composition at high spatial resolution non-invasively in sufficient penetration depth, it paves the way to personalized medicine in pediatric diseases.

## Technologic background

Up to now, ultrasound has played a central role in pediatric diagnostics. Due to its neglectable risk profile and easy availability, it is the diagnostic tool of choice for a variety of different indications [1–4]. As current state of the art ultrasound technologies, such as contrast-enhanced ultrasound (CEUS) do, on the one hand, allow functional flow-based tissue analysis [5, 6], but they essentially require the application of “acoustic” labels on the other. In addition, when compared to new cross-sectional imaging techniques (e.g., PET-CT), ultrasound is limited in visualizing subcellular composition of tissues and therefore diagnostic possibilities. All of this led to the development of new functional and molecular, non-invasive imaging approaches in recent years.

**Fig. 1:**
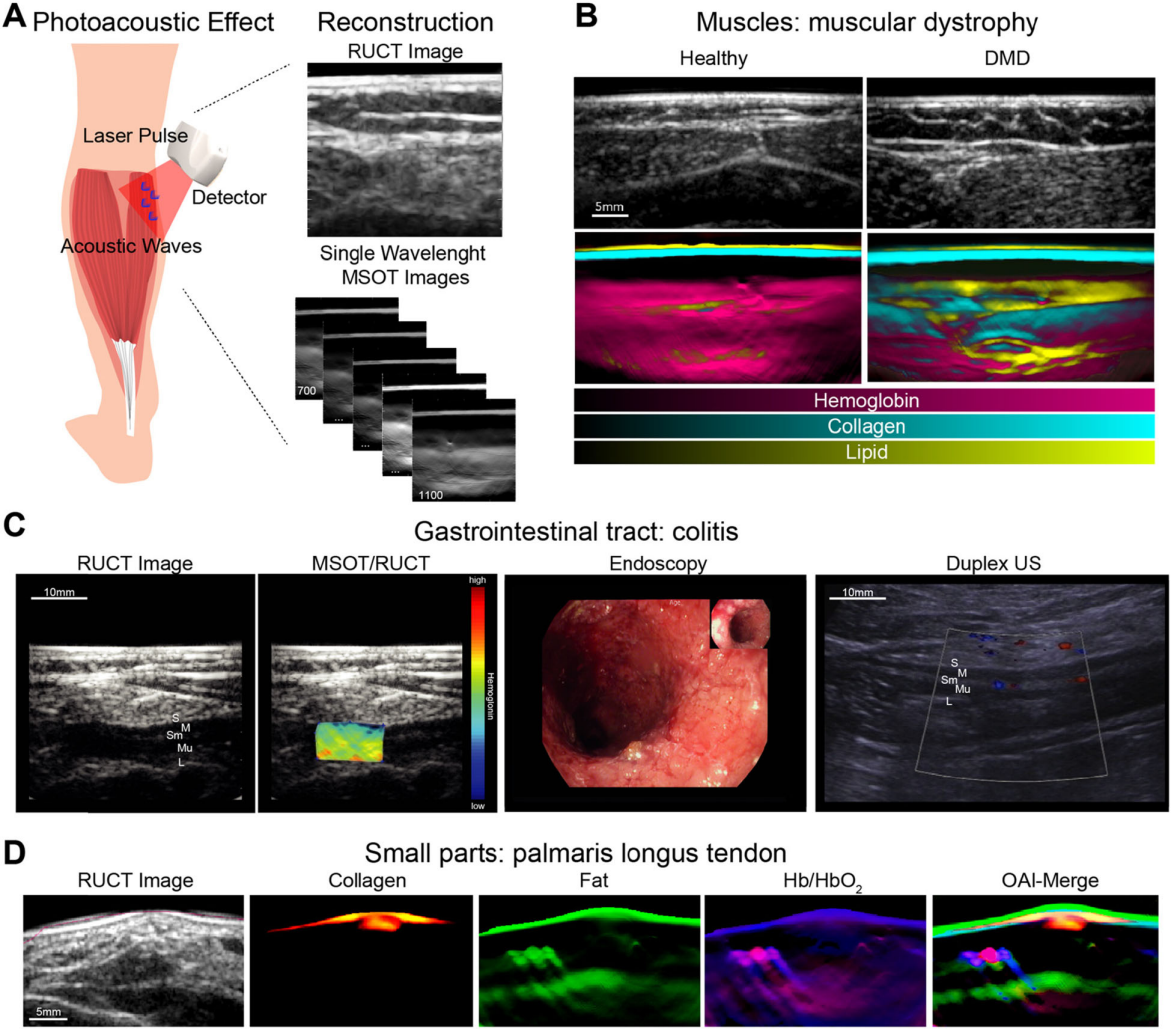
Principle of optoacoustic imaging. **a** The terms “photoacoustic” or “optoacoustic” describe the use of “light” and “sound.” The illumination of tissue by pulsed laser light and the subsequent recording of thermoelastic pressure waves combines the advantages optical (high contrast) and acoustic (high resolution) imaging. Subsequent image reconstruction and spectral unmixing enables non-invasive real-time visualization and quantification of specific endogenous chromophores such as hemoglobin, lipids, and collagens. RUCT reflectance ultrasound computed tomography, MSOT multispectral optoacoustic tomography. **b** Imaging example from muscular imaging resolving hemoglobin, collagen, and fat signals. DMD Duchenne muscular dystrophy. **c** MSOT imaging of the colon unmixed total hemoglobin signal. S serosa, M muscularis mucosa, Sm submucosa, L lumen, US ultrasound. **d** Example for imaging of palmaris longus tendon at the wrist with unmixing for different tissue components. Note the clear delineation of yellow/red containing tendon in comparison of hemoglobin signals from vessels

In this regard, multispectral optoacoustic tomography (MSOT) is rapidly evolving as one promising key technology. MSOT utilizes short-pulsed laser light in the near- and extended near-infrared range (NIR/exNIR) for tissue illumination. As light in these wavelengths has fewer tissue absorption, it enables deeper tissue penetration. The NIR is typically represented in the wavelength between 700 and 900 m followed by extend NIR (exNIR)/NIR II window around 900–1600 nm. In OAI, this light is absorbed by tissue molecules and converted into acoustic pressure waves, which can be recorded and formed into optoacoustic images. In the NIR, endogenous absorbers such as deoxyhemoglobin, oxyhemoglobin, and melanin possess distinct absorption spectra, which gives them a unique signature in MSOT [7, 8]. In the exNIR, further absorption peaks for lipids, water, and collagens can be found [9]. Multispectral imaging, e.g., the utilization of different wavelengths, enables the quantification and spatial resolution of these molecules in MSOT images [10]. A translatable system (MSOT Acuity, iThera Medical GmbH, Munich, Germany) comprises a tunable optical parametric oscillator (OPO) that is pumped by an Nd:YAG laser to provide exact excitation pulses. The pulse energy (30 mJ peak at 730 nm) is as low as to adhere with ANSI limits of maximum permissible exposure (MPE) of the skin. Using different center frequencies, the resolution ranges between 290 μm (4 MHz) and 345 μm (3 MHz), respectively. The same imaging probe can then detect the delivered light and resulting sound waves. The probe is comparable to standard ultrasound systems (Fig. 1). In order to translate the use in clinical scenarios, the technique has been fused with conventional ultrasound imaging, called reflectance ultrasound computed tomography (RUCT) [11]. For RUCT generation, an ultrasound imaging platform is used, which synchronizes ultrasound and optoacoustic image streams [12].

## Current clinical applications

OAI has so far been used in a number of various clinical applications; selected studies are summarized in Table 1. The strength of MSOT is the ability to quantify endogenous chromophores, of which hemoglobin is well known and best absorbing in human tissues. Traditionally, a wavelength of 700  nm is used to detect the peak for deoxygenated hemoglobin (Hb_R_), 800  nm as measure for total hemoglobin (isosbestic point, Hb_T_), and 850  nm for oxygenated hemoglobin (HbO_2_).

**Table 1:** Selected clinical OAI studies

Anatomical region/organ	Chromophores	Wavelength (nm)	Clinical output	References
Non-melanoma skin cancer	Hb_R_, HbO_2,_ melanin	700, 715, 730, 760, 780, 790, 800, 825, 850, 900	Mapping and visualization of skin cancer	[13, 14]
Skin (hair follicles)	Hb_R_, HbO_2_, melanin, lipids	660–1300	Measurement of the structural and physiological features of intact hair follicles	[15]
Vasculature	Hb_R_, HbO_2_	700–850	Noninvasive diagnosis and monitoring of vascular malformations	[16]
	Hb_R_, HbO_2_	700–960	Clinical flow-mediated dilatation measurements	[17]
	HbO_2_. Hb_R_, SO_2_	730, 750, 800, 830	Visualization of blood vessels, microvasculature and the respective oxygen saturation	[18]
	HbO_2_, Hb_R_, melanin	730, 760, 790, 820, 850	Real-time visualization of blood vessels in 3D	[19, 20]
	HbO_2_, Hb_R_, SO_2_	760, 850	Movement correction and artifact-free imaging	[21]
	HbO_2_, Hb_R_, melanin	700, 720, 740, 760, 780, 800, 820, 840, 860	Human angiography	[22, 23]
	HbO_2_, Hb_R_	680–950, 1064	Visualization of human carotid artery	[24]
Sentinel lymph node (SLN)	Melanin, ICG	700, 730, 760, 800, 850, 900	Identification of SLNs	[25]
	Melanin, ICG	700, 730, 760, 800, 850	Metastatic status of lymph nodes	[26]
	Hb, methylene blue	650, 1064	Identification of SLNs	[27]
Inflammatory bowel disease	Hb_T_, HbO_2_, Hb_R_, SO_2_	700, 730, 760, 800, 850, 900	Inflammatory disease activity in Crohn’s disease	[28, 29]
Breast (cancer)	Hb_T_, HbO_2_, Hb_R_, SO_2_	700, 730, 760, 800, 850	Characterization of healthy tissue and malignant lesions	[30]
	HbO_2_, Hb_R_, water, lipid	700–970 (10 nm steps)		[31]
	HbO_2_, Hb_R_	730, 760, 850		[32]
Thyroid	Hb_T_, HbO_2_, Hb_R_, SO_2_, water, lipids	700, 730, 760, 800, 850, 900, 920, 950	Non-invasive diagnostics by tissue characterization	[33]
	–	–	Optical features of thyroid anatomy	[34]
	HbO_2_, Hb_R_, water, lipid	760, 850, 930, 970	Differentiation between malignant and benign thyroidal nodules	[35]
	HbO_2_	1064	Photoacoustic/ultrasound dual imaging of human thyroid cancer	[36]
Systemic sclerosis	Hb_T_, HbO_2_, Hb_R_	700, 730, 750, 800, 850	Disease activity	[37]
Muscle	Hb_T_, HbO_2_, Hb_R_, lipids, collagens	680, 700, 730, 760, 800, 850, 920, 1000, 1030, 1064, 1100	Disease extent in DMD patients	[38]

*Hb*_*R*_ deoxygenated hemoglobin, *HbO*_*2*_ oxygenated hemoglobin, *Hb*_*T*_ total hemoglobin, *SO*_*2*_ MSOT-derived oxygen saturation

When examining vasculature, MSOT is capable of visualizing major blood vessels and microvasculature [18]. As MSOT resolves hemoglobin in its oxygenated and deoxygenated states through optic absorption properties (whereas conventional Doppler ultrasound measures flow), it can also provide images of hemoglobin oxygen saturation and pulsation [18]. Successful studies were performed for mapping human feet blood vessels [18] as well as carotid arteries [24]. The ability to resolve vessels as small as 100 μm in diameter and within 1-cm depth could also aid to diagnose vascular malformations [16].

In a consecutive study, the subcutaneous finger tissue of patients with systemic sclerosis (SSc) provided significantly lower MSOT values for oxygenated hemoglobin as well as total hemoglobin in comparison to healthy controls, reflecting microvascular dysfuntion in SSc as a possible marker of disease activity [37].

Further investigations explored the quantification of hemoglobin in the intestinal wall of patients with Crohn’s disease (CD) with MSOT. These measures have been shown to be increased in patients with active CD, demonstrating robust correlation with endoscopic and microscopic intestinal inflammation [28]. MSOT could therefore be used to differentiate between active disease and remission in affected patients in a non-invasive fashion [29]. Its application is currently further explored in order to develop a clinically (CE) certified technology (www.euphoria2020.eu).

One of the main tissues examined with MSOT is breast tissue. In healthy breast tissue, hormone-related physiological changes of breast parenchyma were visualized with MSOT using only a three wavelengths illumination (700, 800, and 850  nm wavelengths) [39]. The authors found that intensity values were significantly higher at all excitation wavelengths in the secretory compared to the proliferative/follicular phase [39]. In malignant breast tissue, MSOT allowed the visualization of peripheral tumor vascularization, disruption of fat, and water tissue layers [31]. In addition, increased signals for hemoglobin were found in invasive breast cancer when compared to healthy breast tissue [30].

The high resolution in OAI/PAI makes it possible to reveal more vessels than with conventional Doppler as shown in a photoacoustic/ultrasound dual imaging study of human thyroid cancers [36]. While Doppler has sensitivity limits in terms of detecting movements/flow in small vessels, the precise visualization of hemoglobin containing tumor-related bloods vessels could improve cancer diagnosis [40], raising the hope to develop radiation-free screening technologies or treatment monitoring of other (solid) tumors for application in children.

In near-filed applications such as non-melanoma skin cancers MSOT already successfully distinguished skin cancer from normal skin, being helpful for the visualization of lesion margins [13, 14]. Even the detection of metastatic spread of melanoma to sentinel lymph nodes appears to be improved with MSOT when compared to current sentinel lymph node excision protocols [25, 26]. Further, it was demonstrated that the injection of indocyanine green (ICG) and the detection via MSOT could be a proper replacement strategy for scintigraphy approaches [26].

Besides these promising first clinical studies, OAI has similar limitations like ultrasound. Namely, penetration depth of up to several centimeters as well as air, thick bones, body fat, and body hair can significantly influence image quality in adults. Therefore, children and adolescents might be excellent candidates for MSOT imaging as their organs and/or muscles are closer to the body surface.

Consequently, the first pediatric study showed that MSOT imaging is able to visualize the molecular composition of muscles in children. Muscular collagen content in children with Duchenne muscular dystrophy was increased as compared to healthy controls, suggesting non-invasive measured collagen content as a novel non-invasive biomarker for disease severity and potential monitoring tool for novel therapies [38]. Given the rise of genetic therapies, these findings underline the potential of MSOT for further personalized molecular imaging applications in children. A great advantage is that MSOT imaging can be performed without sedation or general anesthesia, which is especially important in patients with muscular disorders with respiratory depression. By using MSOT imaging, a substantial number of invasive procedures could hopefully be saved in the future.

## Limitations

For translation into clinical practice, there are still several challenges to overcome: firstly, standardization of OAI imaging is required. It was already reported that preclinical OAI imaging systems consistently perform equal or even better (with variations smaller than 10%) compared to other preclinical imaging modalities, which underlines their clinical potential [41]. In agreement with these findings, a small clinical study suggests that clinical MSOT provides consistent and reproducible results in humans as well [42]. For further validation, besides comparisons with other cross-sectional imaging modalities (US/MRI), prospective clinical trials with higher case number and multi-centric approaches with clinically meaningful outcomes are required. With the International Photoacoustic Standardization Consortium (IPASC), first efforts are currently made to facilitate an international debate and consensus to address this need [43]. Currently, there are two CE-marked OAI systems for clinical use: Imagio by Seno Medical Instruments and MSOT Acuity by iThera Medical GmbH. The high costs for OAI systems, especially when compared to current high-end ultrasound systems, are a main challenge for clinical implementation. Various approaches for reducing these costs are already in discussion, including alternate illumination sources and signal detection methods [44]. A higher number of commercially available systems might also help to reduce costs and allow a widespread adoption of MSOT in clinical practice.

## Future perspective

MSOT is capable of visualizing endogenous chromophores such as hemoglobin, melanin, lipids, and recently collagens. Hemoglobin and collagens in particular display specific properties that are promising for monitoring the course of various diseases. Furthermore, the use of exogenous chromophores (e.g., optoacoustic targeted therapies) as contrast agents for OAI imaging in pediatric patients is still unexplored. Therefore, the combination of endogenous and exogenous molecular information could pave the way for novel tailored theranostic approaches in pediatrics in the future.

## Data Availability

Not applicable.

## Abbreviations

CD: Crohn’s disease
DMD: Duchenne muscular dystrophy
exNIR: Extended near-infrared range
HBO_2_: Oxygenated hemoglobin
Hb_R_: Deoxygenated hemoglobin
Hb_T_: Total hemoglobin
MPE: Maximum permissible exposure
MSOT: Multispectral optoacoustic tomography
NIR: Near-infrared range
OAI: Optoacoustic imaging
RUCT: Reflectance ultrasound computed tomography
SaO_2_: MSOT-derived oxygen saturation
